# Data on hyper-activation of GPVI signalling in obese patients: Towards the identification of novel antiplatelet targets in obesity

**DOI:** 10.1016/j.dib.2019.103784

**Published:** 2019-02-28

**Authors:** María N. Barrachina, Irene Izquierdo, Lidia Hermida-Nogueira, Aurelio M. Sueiro, Esteban Guitián, Felipe F. Casanueva, Richard W. Farndale, Masaaki Moroi, Stephanie M. Jung, María Pardo, Ángel García

**Affiliations:** a Platelet Proteomics Group, Center for Research in Molecular Medicine and Chronic Diseases (CIMUS), Universidade Santiago de Compostela, Spain; b Instituto de Investigación Sanitaria de Santiago (IDIS), Spain; c Grupo de Endocrinología Molecular y Celular, Instituto de Investigación Sanitaria de Santiago (IDIS)/ Servicio de Endocrinología, Xerencia de Xestión Integrada de Santiago (XXS), Spain; d Mass Spectrometry and Proteomic Unit, Rede de Infraestructuras de Apoio á Investigación e ao Desenvolvemento Tecnolóxico, Universidade de Santiago de Compostela, Santiago de Compostela, Spain; e Department of Biochemistry, University of Cambridge, Cambridge, CB2 1QW, United Kingdom; f Grupo Obesidómica – Instituto de Investigación Sanitaria de Santiago (IDIS)

## Abstract

This data article is associated with the manuscript “GPVI surface expression and signalling pathway activation are increased in platelets from obese patients: elucidating potential anti-atherothrombotic targets in obesity” [1]. The study refers to a combination of different approaches in order to identify platelet-derived biomarkers in obesity. A total of 34 obese patients and their lean-matched controls were included in the study. We carried out a proteomic and functional (aggregation assays) analysis to find alterations in platelet-derived signalling pathways. After that, biochemical and mechanistic (flow cytometry assays) approaches were done in order to confirm a hyperactivation of the GPVI-related signalling pathway.

**Table:** Specifications Table

Subject area	*Biology, Biochemistry*
More specific subject area	*Proteomics, Mass spectrometry, Biochemistry*
Type of data	*Tables and figures*
How data was acquired	*Two-dimensional differential in-gel electrophoresis (2D-DIGE), Mass spectrometry, Flow Cytometry (Accuri C6 flow cytometer), Immunoprecipitation, Aggregation assays (Chrono-log aggregometer), Western blot, System biology (Ingenuity Pathways Analysis (IPA) and STRING)*
Data format	*Analyzed*
Experimental factors	*Protein extraction and digestion for proteomics, biochemical and functional analysis.*
Experimental features	*2D-DIGE coupled to mass spectrometry; western blot assays; aggregation assays; flow cytometry assays.*
Data source location	*Platelet Proteomics Group. Center for Research in Molecular Medicine and Chronic Diseases (CIMUS), Universidade Santiago de Compostela*
Data accessibility	*Data are presented within this article and related to an original research article (in press)*

**Table:** 

**Value of the data** • A combination of proteomics, biochemical and functional approaches was carried out in order to obtain platelet-derived biomarkers and drug targets in obesity • The dataset can be utilized for comparative analysis in platelets between obese and lean individuals • The present data illustrates how the combination of proteomics and functional analyses could elucidate potential anti-atherothrombotic targets in platelet-related diseases

## 1 Data

**Fig. 1:**
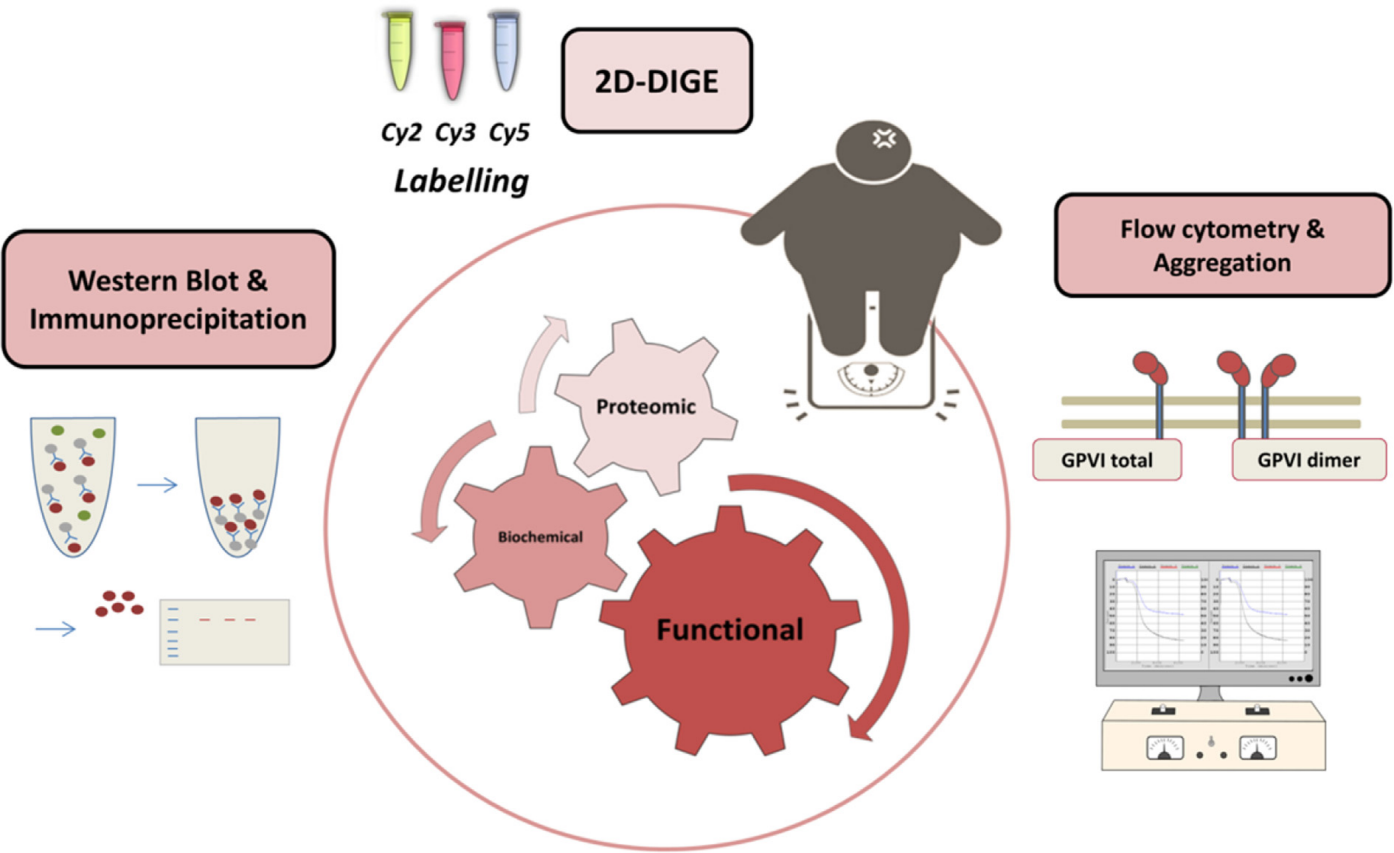
Schematic workflow of the study.

We performed a combination of 2D-DIGE-based proteomics, biochemical and functional approaches in order to identify biomarkers related to the risk of suffering atherothrombosis in obese patients without cardiovascular disease (Fig. 1). Regarding the proteomic analysis, 1895 protein spots were detected per gel, 55 of which were differentially regulated between obese and lean groups (fold change cut-off ≥ 1.2; p < 0.05). We could successfully identify 35 protein features by MS which corresponded to 33 different ORFs (Table 1). Among others, we confirmed an up-regulation of αIIb and fibrinogen isoforms in platelets from obese patients. Besides, a complementary platelet aggregation approach showed platelets from obese patients are hyper-reactive in response to collagen and collagen-related peptide (CRP), revealing the collagen receptor Glycoprotein VI (GPVI) signalling as one of the altered pathways. We also found the active form of Src (pTyr418) is up-regulated in platelets from obese individuals, which links proteomics and aggregation data. Moreover, we show that CRP-activated platelets present higher levels of tyrosine phosphorylated PLCγ2 in obese patients, confirming alterations in GPVI signalling. In line with the above, flow cytometry studies show higher surface expression levels of total GPVI and GPVI-dimer in obese platelets, both correlating with BMI.

**Table 1:** Platelet proteins differentially regulated in obese patients versus lean healthy controls.

Uniprot code	Name	Spot	Fold change
ACTB_HUMAN	Actin, cytoplasmic 1	4405	+1.3
		4325	+1.3
		2021	+1.2
		2550	+1.2
		4461	+1.2
ACTG_HUMAN	Actin, cytoplasmic 2	2550	+1.2
		4461	+1.2
ACTN1_HUMAN	Alpha-actinin-1	4465	+1.2
		4405	+1.3
		4403	+1.3
ALBU_HUMAN	Serum albumin	4424	−1.2
		4356	+1.3
ANXA5_HUMAN	Annexin A5	4356	+1.3
ARK72_HUMAN	Aflatoxin B1 aldehyde reductase member 2	2898	−1.2
DCTN2_HUMAN	Dynactin subunit 2	4354	−1.2
DPYL2_HUMAN	Dihydropyrimidinase-related protein 2	1858	−1.2
FIBB_HUMAN	Fibrinogen beta chain	2547	+1.2
		2077	+1.2
FIBG_HUMAN	Fibrinogen gamma chain	4465	+1.2
		4325	+1.3
		2232	+1.3
		4304	+1.2
		4344	+1.2
GELS_HUMAN	Gelsolin	4508	−1.2
		4505	−1.2
GPDM_HUMAN	Glycerol-3-phosphate dehydrogenase, mitochondrial	4400	−1.4
HEM2_HUMAN	Delta-aminolevulinic acid dehydratase	2932	−1.2
HSPB1_HUMAN	Heat shock protein beta-1	4361	−1.2
		3287	−1.2
ITA2B_HUMAN	Integrin αIIb	993	+1.2
		1006	+1.2
LYSC_HUMAN	Lysozyme C	4320	−1.3
ODO2_HUMAN	Dihydrolipoyllysine-residue succinyltransferase component of 2-oxoglutarate dehydrogenase complex, mitochondrial	2077	+1.2
PDLI1_HUMAN	PDZ and LIM domain protein 1	2782	−1.2
PDIA6_HUMAN	Protein disulfide-isomerase A6	4354	−1.2
PGM2_HUMAN	Phosphoglucomutase-2	1711	−1.2
PI42A_HUMAN	Phosphatidylinositol 5-phosphate 4-kinase type-2 alpha	4359	−1.2
PP1R7_HUMAN	Protein phosphatase 1 regulatory subunit 7	4465	+1.2
SEPT2_HUMAN	Septin-2	2547	+1.2
SEP11_HUMAN	Septin-11	4359	−1.2
STIP1_HUMAN	Stress-induced-phosphoprotein 1	1858	−1.2
TBB1_HUMAN	Tubulin beta-1 chain	4354	−1.2
TPM1_HUMAN	Tropomyosin alpha-1 chain	4424	−1.2
		4348	−1.2
TSP1_HUMAN	Thrombospondin-1	993	+1.2
		4405	+1.3
		4321	+1.2
		4311	+1.2
		4403	+1.3
TXNL1_HUMAN	Thioredoxin-like protein 1	2867	+1.2
TYPH_HUMAN	Thymidine phosphorylase	2021	+1.2
VINC_HUMAN	Vinculin	933	+1.2
		1006	+1.2
VP37B_HUMAN	Vacuolar protein sorting-associated protein 37B	2932	−1.2
ZYX_HUMAN	Zyxin	4491	−1.2
		4339	−1.3

^a^. A positive fold change indicates that the protein feature is up-regulated in obese patients, whereas a negative fold change indicates that the spot is down-regulate.

## 2 Experimental design, materials and methods

A detailed description of the material and methods used is provided in the accompanying research article [1]. Essential methodical information related to the present data is provided in the following sections.

### 2.1 Patients

The study was approved by the local Ethics Committee (Galician Investigation Ethics Committee) and developed according to the principles outlined in the Declaration of Helsinki. All methods were carried out in accordance with the approved guidelines, and written informed consent was obtained from all subjects. At the moment of diagnosis, the obese patients were asked to participate in the study. In the case of acceptance, they signed the informed consent, and 36 mL of blood were collected in sodium citrate Vacuette^®^ tubes for analyses.

Thirty-four patients referred to the Endocrinology Unit at the Santiago de Compostela University Hospital, Spain, participated in the study. These patients had a BMI higher than 40, indicating severe “healthy” obesity, and were referred to hospital by the primary care physician so they could be treated by an endocrinologist. Exclusion criteria were coagulation disorders, platelet-associated disorders, chronic antiplatelet drugs and other chronic drug therapy (except for drugs required to treat pre-existing clinical factors that do not affect platelet reactivity). The lean control group consisted of healthy normal weight volunteers recruited at the Santiago de Compostela University Health Service. This group was age- and gender-matched with the obese group and individuals had a BMI between 18 and 26.

Additionally, another group of 11 healthy volunteers with BMI between 26 and 35 (overweight and grade 1 obese individuals) was included in the study in order to investigate the expression levels of GPVI in the platelet surface. This group, age-matched with the other groups, was also recruited at the Santiago de Compostela University Health Service.

### 2.2 Platelet isolation

Fresh blood samples (36 mL) were collected from obese patients and lean matched-controls in coagulation 3.2% sodium citrate Vacuette^®^ tubes and processed in less than 60 min. In order to obtain the platelet-rich plasma (PRP), blood was centrifuged for 20 min at 200 g at room temperature.

Washed platelets were isolated following an established method that limits blood cells or plasma proteins contaminations [2]. Briefly, upon addition of warmed (30 °C) acid citrate dextrose (ACD) solution (117 mM sodium citrate, 111 mM glucose, 78 mM citric acid) to a concentration of 7% v/v, the blood was centrifuged for 20 min at 200×*g* at room temperature to obtain PRP. The upper two thirds of the PRP was centrifuged in a fresh tube at 1000g in the presence of prostacyclin (final concentration 0.2 μg/mL), which allowed pelleting of platelets. Following removal of plasma, platelets were washed in Tyrodes-HEPES (134 mmol/L NaCl, 0.34 mmol/L Na_2_HPO4, 2.9 mmol/LKCl, 12 mmol/L NaHCO_3_, 20 mmol/L HEPES, 5 mmol/L glucose, 1 mmol/L MgCl_2_, pH 7.3). Final washed platelet pellets were resuspended in HEPES-Tyrodes (134 mmol/L NaCl, 0.34 mmol/L Na_2_HPO_4_, 2.9 mmol/L KCl, 12 mmol/L NaHCO_3_, 20 mmol/L HEPES, 5 mmol/L glucose, 1 mmol/L MgCl_2_, pH 7.3) and allowed to rest for 30 minutes at room temperature. For proteomic studies based on two-dimensional differential gel electrophoresis (2D-DIGE), concentrations of 8 × 10^8^ platelets/mL were lysed in a NP-40-based lysis buffer (0.3 M Sodium Chloride, 20mM Tris, 2mM EGTA, 2mM EDTA, 2% (v/v) NP-40, pH 7.5) and stored at −80 °C [3].

### 2.3 Aggregation approach

PRP or washed platelets (2.5 × 10^8^ platelets/mL in HEPES-Tyrodes) were used for aggregation studies. Aggregations were performed with 300μL aliquots of PRP or washed platelets that were warmed at 37 °C for 4 min without stirring and for 1 min with constant stirring at 1200 rpm in a Chrono-log aggregometer, before stimulation with the corresponding agonists for 6 min.

The following agonists were used: Crosslinked collagen-related peptide (CRP), with the sequence Gly-Cys-Hyp-(Gly-Pro-Hyp)10-Gly-Cys-Hyp-Gly-NH2, was provided by Dr. Richard W. Farndale, from the University of Cambridge (UK). Collagen Reagent HORM^®^ Suspension (KRH) was purchased from Takeda Austria GmbH (Austria). Thrombin and ADP were purchased from Sigma (Sigma-Aldrich, St. Louis, MO) and arachidonic acid from Cayman Chemical (Michigan, USA). Rhodocytin was provided by Johannes A. Eble, from Center for Molecular Medicine, Excellence Cluster Cardio-Pulmonary System, Frankfurt University Hospital, Frankfurt am Main, Germany.

Regarding the activating condition, PRP stimulations were done with collagen-related peptide (CRP)-XL (0.1, 0.15 and 0.2 μg/mL), Horm collagen (0.5, 0.75 and 1 μg/mL), rhodocytin (25 and 50nM), ADP (2 and 3 μM) and arachidonic acid (0.3 and 0.5mM). On the other hand, washed platelets were activated with collagen-related peptide (CRP)-XL (0.4, 0.5 and 1 μg/mL), Horm collagen (1, 2 and 3 μg/mL), rhodocytin (25, 50 and 100nM) and thrombin (0.5 and 0.75 U/mL).

### 2.4 2D-DIGE approach

For proteomic analysis, proteins were precipitated in 20% trichloroacetic acid in acetone, as previously described [2]. Protein pellets were resuspended in 60 μL DIGE buffer (65 mM CHAPS, 5 M urea, 2 M thiourea, 0.15 M NDSB-256, 30 mM Tris, 1 mM sodium vanadate, 0.1 mM sodium fluoride, and 1 mM benzamidine). Protein quantitation was done with Coomassie Plus protein reagent (Thermo Scientific).

Six gels (technical replicates) were run in the experiment with a total of 150 μg of mixed labeled protein per gel. These protein mixtures contained 50 μg of protein from each sample (10 obese pooled and 10 lean pooled matched-controls) randomly labeled with 400 pmol minimal CyDye DIGE fluors (Cy3 and Cy5), and 50 μg of a pool of both conditions (25 μg from obese patients and 25 μg from lean controls) labeled with 400 pmol Cy2 (internal standard). Labelling was performed for 30 min on ice in the dark. The reaction was stopped with 1 μl of 10 mM lysine acting for 10 min on ice in the dark. After labelling step, the three samples were pooled and an equal volume of 2 × sample buffer was added (65 mM CHAPS, 2 M thiourea, 5 M urea, 0.15 M NDSB-256, 130 mM DTT, 4 mM tributylphosphine, 1 mM sodium vanadate, 0.1 mM sodium fluoride, and 1 mM benzamidine).

After mixing, the tube was left for 10 min on ice in the dark. For reswelling, samples were diluted up to a total of 500 μl of 2D Sample buffer (5 M urea, 2 M thiourea, 2 mM tributylphosphine, 65 mM DTT, 65 mM CHAPS, 0.15 M NDSB-256, 1 mM sodium vanadate, 0.1 mM sodium fluoride, and 1 mM benzamidine, final concentration), and ampholytes (Servalyt 4–7) were added to a final concentration of 1.6% (v/v). IPG strips were rehydrated with the samples for 16 h in the dark. Isoelectric focusing (IEF) was run on 24 cm, pH 4–7 IPG strips (GE Healthcare) powered by a Multiphor II (GE Healthcare) for 64.9 kVh at 17 °C.

Following the first dimension, IPG strips were immediately equilibrated for 15 min in reduction buffer (6 M urea, 50 mM tris pH 8.8, 30% glycerol, 2% w/v SDS, 65 mM DTT and traces of bromophenol blue) and then for 15 min in alkylation buffer (6 M urea, 50 mM tris pH 8.8, 30% glycerol, 2% w/v SDS, 135 mM iodoacetamide and traces of bromophenol blue) with gentle agitation; all steps in the dark. IPG strips were washed out with ultrapure water and placed on top of the second dimension gels, embedded with 0.5% melted agarose.

Proteins were separated in the second dimension by SDS-polyacrylamide gel electrophoresis (PAGE) on 11% polyacrylamide gels at run conditions of 10 °C, 20 mA per gel for 1 h, followed by 40 mA per gel for 4 h by using an Ettan Dalt 6 system (GE Healthcare). Following electrophoresis gels were scanned directly in a Typhoon FLA 7000 scanner (GE Healthcare). After imaging, gels were fixed in 10% methanol/7% acetic acid for 1 hour, and stained overnight with Sypro Ruby fluorescent dye for spot picking.

### 2.5 Differential image analysis

Scanned images were processed with Progenesis SameSpots (Version 4.5) from Nonlinear Dynamics. Manual and automatic alignment was used to align the images. SameSpots detects the spots simultaneously across all images generating a master gel list containing all the features. Internal calibration of the 2D-DIGE gel images with regard to pI and molecular weight was carried out with SameSpots built-in tools. All gels were compared with each other, and fold values as well as P values of all spots were calculated by SamesSpots Version 4.5 software using 1-way ANOVA analysis. Differential expression of a protein present in the gels was considered significant when the fold change was at least 1.2 and the P value was <0.05.

### 2.6 Mass spectrometric analysis

Differential regulated spots were excised manually from the gels and in-gel digested with trypsin as previously indicated [4]. Most of the identifications were by LC–MS/MS on an EASY-nLC (Proxeon, Bruker Daltonics) and a Bruker Amazon ETD ion trap. Remaining identifications were by MALDI-TOF(/TOF), in a 4800 Plus MALDI-TOF/TOF analyzer (Applied Biosystems).

For LC-MS/MS analysis, digested peptide mixtures were dissolved in 0.1% formic acid and were separated in an EASY-nLC (Proxeon, Bruker Daltonik GmbH) with a reverse phase nanocolumn (Easy column SC200) from Proxeon (see below for more details).

**Table:** Table: LC parameters for separation of tryptic peptides

LC settings	
*LC system*	Easy-nLC PROXEON
*Trap column*	Easy-column SC001 L 2 cm, ID 100 μm, 5 μm, 120 A, C18-A1 from Proxeon
*Analytical column*	Easy column SC200C18 3μm 120A 360 μm OD/75μm ID, L = 10cm) from Proxeon
*Flow rate*	300 nl/min
*Eluents*	A:0.1% FA in water B:0.1%FA in ACN
*Gradient*	5% (t = 0 min), 35% (t = 32 min), 50% (t = 37 min), 100% (t = 38min)

Ionized peptides were analyzed in a CID-ETD ion trap mass spectrometer (Bruker Daltonics), equipped with a Nanosprayer ionization source that was used for data-dependent MS/MS experiments. Spectra were acquired in Enhanced Resolution mode. Further acquisition parameters are listed in below.

**Table:** Table: MS and MS/MS settings used for acquisition with the Amazon ETD

*Acquisition parameters*	
*Source*	Nanosprayer^®^
*MS settings:*	
*Scan mode*	Enhanced resolution mode (8100 *m*/*z* s^−1^)
*Scan range*	50–3000 Da
*Spectra averages*	5 (Rolling averaging: 1)
*MS/MS settings:*	
*Scan mode*	UltraScan
*Scan range*	100-3000 *m*/*z*
*No. of precursor ions*	3 (active exclusion after 1 spectrum, release after 0.2 min; reconsider precursor, if current intensity/previous intensity >1%
*Isolation width*	4 *m*/*z*
*Spectral averages*	2
*Fragmentation amplitude (CID)*	30–300%
*Fragmentation time (ETD)*	100 ms

Automated analysis of mass data was achieved by Data Analysis 4.0 and BioTools 3.2 from Bruker Daltonik GmbH. Database search was performed with the Mascot v2.3.0 search tool (Matrix Science, London, UK) screening SwissProt (SwissProt_2016_09.fasta). Searches were restricted to human taxonomy allowing carbamidomethyl cysteine as a fixed modification and oxidized methionine as potential variable modification. Both the precursor mass tolerance and the MS/MS tolerance were set at 0.3 and 0.4 Da, respectively, allowing 1 missed tryptic cleavage site. All spectra and database results were manually inspected in detail using the above software, especially in the case of identifications based on one peptide hit. For the latter, positive identification by MS was only accepted when more than 50% y-ions (CID fragmentation) or z-ions (ETD fragmentation) were obtained for a peptide comprising at least eight amino acids long and no missed tryptic cleavage site. Positive hits corresponded to Mascot scores >40 plus the fulfillment of the above criteria.

For MALDI analysis, dried peptides were dissolved in 4 μL of 0.5% formic acid. Equal volumes (0.5 μL) of peptide and matrix solution (3 mg alpha-cyano-4-hydroxycinnamic acid (α-CHCA) dissolved in 1 mL of 50% acetonitrile in 0.1% trifluoroacetic acid) were deposited using the thin layer method, onto a 384 Opti-TOF 123 × 81 mm MALDI plate (Applied Biosystems) and allowed to dry at room temperature. Mass spectrometric data were obtained in an automated analysis loop using a 4800 MALDI-TOF/TOF analyzer (Applied Biosystems). Spectra were acquired in the reflector positive-ion mode with a Nd:YAG, 355nm wavelength laser, at 200 Hz laser frequency, and 1000 to 2000 individual spectra were averaged. The experiments were acquired uniform with fixed laser intensity. All MSMS spectra were performed by selecting the precursors with a relative resolution of 300 (FWHM) and metastable suppression.

Automated analysis of mass data was achieved by using the 4000 Series Explorer Software V3.5 (Applied Biosystems). Internal calibration of MALDI-TOF mass spectra was performed using two trypsin autolysis ions with *m*/*z* = 842.510 and *m*/*z* = 2211.105. For MALDI-MS/MS, calibrations were performed with fragment ion spectra obtained for Angiotensin II (peptide mix, calibration standard, Bruker). MS and MS/MS spectra data were combined through the Protein Pilot Explorer Software v4.5. Database search was performed with the Mascot v2.1 search tool (Matrix Science, London, UK) screening SwissProt (release version 2016–05; February 551193 entries). Searches were restricted to Human taxonomy allowing carbamidomethyl cysteine as a fixed modification and oxidized methionine as potential variable modification. Both the precursor mass tolerance and the MS/MS tolerance were set at 100 ppm and 0.3 Da, respectively, allowing 1 missed tryptic cleavage site. MALDI-MS(/MS) spectra and database search results were manually inspected in detail using the previous software. For combined MS and MS/MS data, identifications were accepted when Confidence Interval (C.I.%) of Protein Pilot software was 95% or higher. Since Protein Scores and Ion Scores from different searches cannot be directly compared, Protein Pilot software calculates this C.I.% in order to combine results from MS and MS/MS database searches. This coefficient value means that the probability that the observed match is a random event is lower than 5%. For PMF spectra, identifications were also accepted when (C.I.%) of Protein Pilot software was 99% or higher.

### 2.7 Western blotting

Western blotting was performed for validation purpose. 1D-western blotting was carried out to validate individual samples (biological replicates). 11% SDS-PAGE gels were used, loading 10 μg of protein per lane. For 2D-western blotting validations, IPG strips 4–7, 7 cm (GE Healthcare), were used. Thirty micrograms of protein were loaded per strip; obese and lean-matched samples (pools of 5 individuals per each condition) were analyzed in parallel. Samples were analyzed in triplicate. IEF (first dimension) was for 11Kvh; second dimension was also on 11% SDS-PAGE gels.

Following electrophoresis, proteins were transferred onto polyvinylidene difluoride (PVDF) membranes. Membranes were blocked in 5% BSA in TBS-T (20 mM Tris-HCl, (pH 7.6), 150 mM NaCl and 0.1% Tween 20) overnight at 4 °C and incubated for 90 min at room temperature with the following primary antibodies: rabbit anti-phospho-SRC (Tyr418) (Invitrogen), dilution 1:1000; rabbit anti-SRC pan (Invitrogen), dilution 1:1000; mouse anti-FIB (sc-69775, Santa Cruz), dilution 1:1000; mouse anti-PLCγ2 (sc-5283, Santa Cruz), dilution 1:1000; rabbit anti-GAPDH (SIGMA), dilution 1:5000; and anti-G6f (1:1000), produced by CovalAB UK (Cambridge, UK). Following washes in TBS-T, membranes were exposed to horseradish peroxidase–labeled goat anti-rabbit, or goat anti-mouse antibodies (dilution 1:5000) (Pierce, Rockford, IL), and processed using an enhanced-chemiluminiscence system (ECL, Pierce, Rockford, USA).

### 2.8 Immunoprecipitation

Basal and activated platelets (8 × 10^8^platelets/mL, 500 μL per immunoprecipitation; activations with CRP-XL 1 μg/mL, 90sec) were lysed with 500 μL NP40-based lysis buffer, (0.3 M Sodium Chloride, 20mM Tris, 2mM EGTA, 2mM EDTA, 2% (v/v) NP-40, pH 7.5). Activations were under non-aggregating conditions in the presence of integrilin (9 μM). For phosphotyrosine (*p*-Tyr) immunoprecipitations, 5 μg of agarose-conjugated 4G10 monoclonal anti-phosphotyrosine antibody (EMD Millipore Corporation, Billerica, MA, USA) were added to the lysates per immunoprecipitation and samples rotated overnight at 4 °C. Before the addition of the antibodies, samples were precleared with 25 μL of Protein A-Sepharose (50% w/v in TBS-T (20mM Tris-HCl (pH 7.6), 137mM NaCl, and 0.1% v/v Tween 20)) at 4 °C for 60 minutes with end-over-end mixing. After immunoprecipitations, proteins were eluted from the beads in 2X Laemmli sample buffer (4% w/v SDS, 10% v/v 2-mercaptoethanol, 20% v/v glycerol, 50 mM Tris, pH 6.8) and resolved on 4–12% NuPAGE Bis-Tris gradient gels (Invitrogen, Carlsbad, CA, USA) for western blotting.

### 2.9 Flow cytometry

We analyzed the expression of GPVI in whole blood and P-selectin in washed platelets. To measure GPVI dimer, GPVI total and P-selectin, 10 μl of platelet solution, either 5-fold diluted whole blood or washed platelets (5 × 10^7^ cells/mL), was mixed with 10 μl of 204–11 Fab (GPVI-dimer-specific; 5 μg/mL, final), 1G5 (pan GPVI; 10 μg/mL, final) (Biocytex, France) and CD62 (10 μg/mL, final) (ABCAM, UK) and incubated for 10 min. For all of them, ALEXA 488 anti-mouse F(ab)2 antibody (50 μg/ml, final) (Jackson ImmunoResearch) was added as the secondary antibody and incubated for 10 min. In individual Eppendorf tubes each reaction mixture was diluted with 0.200 ml of diluent (Beckman Coulter, USA), and then antibody binding was measured by an Accuri C6 flow cytometer (BD Biosciences). Platelet binding to appropriate controls, mouse Fab (Jackson ImmunoResearch) when the primary antibody was 204–11 Fab, IgG2A when it was 1G5 and IgG1 when it was CD62, was determined. The test was run in triplicate and the average of mean fluorescence intensity was measured.

### 2.10 Systems biology

Ingenuity Pathways Analysis software (IPA, Ingenuity Systems, www.ingenuity.com) was used to investigate possible interactions among all the identified proteins. Interactive pathways were generated to observe potential direct and indirect relations among the differentially regulated proteins. STRING v10 software [5] was also used to predicted protein-protein interactions and to know the biological process, molecular functions and cellular components where the differential proteins were involved.

### 2.11 Statistical analysis

Categorical variables from obese patients and lean matched-controls are expressed as percentages and were compared using the Fisher exact test. Continuous variables are expressed as the median ± SD, and were compared by the Mann-Whitney test. Correlations analyses were done using the Spearman's test.

As indicated above, the differential proteomic analysis was done analyzing all the spots between obese patients and lean matched-controls; for a given spot, the probability value was calculated using the quantified and normalized volumes for the matched spot. All probability values were calculated using 1-way ANOVA analysis and values of p < 0.05 were considered statistically significant.

Following 1D-western blotting validation studies, densitometry of the bands were performed using ImageJ (National Institute of Health, Bethesda, MD, USA) version 1.47, and statistical analysis by Mann–Whitney test comparing groups. Graphs were done with GraphPad Prism 5 (GraphPad Software, Inc. San Diego California, USA). All analyses were performed using IBM SPSS Statistics 20 software for Windows (IBM, Armonk, NY, USA).
